# Perianal and perineal rhabdomyosarcomas: a retrospective multicenter study of 35 cases

**DOI:** 10.1186/s12893-021-01073-x

**Published:** 2021-01-30

**Authors:** Yaoyu Guo, Bang Hu, Dandan Huang, Xinhua Wang, Juan Li, Di Zhang, Xueying Li, Gong Chen, Donglin Ren

**Affiliations:** 1 The Sixth Affiliated Hospital, Sun Yat-Sen University, 26 Yuancun Erheng Road, Tianhe District, Guangzhou, 510655 People’s Republic of China; 2 Guangdong Provincial Key Laboratory of Colorectal and Pelvic Floor Diseases, 26 Yuancun Erheng Road, Tianhe District, Guangzhou, 510655 People’s Republic of China; 3 Department of Colorectal Surgery, Sun Yat-Sen University Cancer Center, 651 Dongfeng East Road, Yuexiu District, Guangzhou, 510080 People’s Republic of China

**Keywords:** Perianal, Perineal, Abscess, Rhabdomyosarcoma

## Abstract

**Background:**

Perianal/perineal rhabdomyosarcomas (PRMS) are easily misdiagnosed soft tissue tumours with a poor prognosis. This study was designed to analyze the clinical, diagnostic, pathological and prognostic features of PRMS, and to explore currently available therapeutic modalities.

**Methods:**

Clinical data of PRMS patients admitted to the Sixth Affiliated Hospital and the Cancer Center of Sun Yat-sen University and from related Chinese literature published from 1987 to 2018 were collected and analyzed. The Chi-square test was used to evaluate the differences between each group. The Kaplan–Meier methods were applied to estimate and compare survival rates.

**Results:**

A total of 35 patients were included in this study; 20 identified within related Chinese literatures and 15 from our center admitted during the period of 1997–2019. Out of these cases, 34 presented with perianal masses and the remaining one manifested as an inguinal mass. Moreover, 20 patients complained of pain and 16 of them were misdiagnosed as perianal abscesses, in which the presence of pain contributed to the misdiagnosis (p < 0.05). The average time interval between symptom onset and pathological diagnosis was 3.1 months. Next, 13 cases were classified into IRS group III/IV and 20 cases into stages 3/4. Additionally, 14 and 9 cases received the pathological diagnoses of embryonal rhabdomyosarcoma and alveolar rhabdomyosarcoma, respectively. Regarding the patients’ survival rates, five patients survived for more than 2 years, and three of them survived for more than 5 years. The overall 2 years and 5 years survival rates were 32% and 24%, respectively. The symptom of pain and misdiagnosis both contributed to the poor prognosis in these patients (p < 0.05). MRI showed that the PRMS were closely related to external anal sphincter in 10 cases.

**Conclusion:**

PRMS are easily misdiagnosed lesions, which often leads to an unfavourable outcome in affected patients. Patients with painful perianal masses should be evaluated to exclude PRMS. MRI revealed that PRMS are closely related to the external anal sphincter. Multidisciplinary management is recommended in the treatment of PRMS.

## Background

Rhabdomyosarcomas (RMS) are common soft tissue malignancies, but primary perianal and perineal rhabdomyosarcomas (PRMS) are extremely rare, accounting for only 2% of all RMS [1]. PRMS are routinely misdiagnosed and mistreated, associated with a relatively high mortality rate. In this study, 35 cases were reviewed to clarify the clinical manifestations, imaging findings, pathologic and prognostic features of PRMS, as well as to explore the optimal therapeutic regimen for this condition.

## Methods

### Patient eligibility

Fifteen PRMS patients treated and followed up at the Sixth Affiliated Hospital (n = 6) and the Cancer Center (n = 9) of Sun Yat-sen University were reviewed in this study. The perianal region consists of the area posterior or lateral to the anus. The perineal region is clinically defined as the area between the anus and the scrotum or labia magna. Patients with gluteal or deep pelvic primaries involving the perineum or of urogenital origin were excluded [2]. Patients’ baseline information was gathered and analyzed, including their gender, age at first diagnosis, symptoms, preoperative tumor size, lesion pathological classification, lymph node involvement, metastasis and treatment received. Postoperative staging and grouping were in accordance with the IRS (Intergroup Rhabdomyosarcoma Study) standard [3]. A database of medical journals published in Chinese between 1987 and September 2018 was examined and 20 patients were identified [4–19]. Finally, a total of 35 patients were included in this study. This study obtained approval from the Ethics Committee of the Sixth Affiliated Hospital.

### Statistical analysis

The Spss 26.0 software package was used for statistical analysis. The Chi-square test was used to assess the differences between each group. Kaplan–Meier methods were used to assess the Overall Survival (OS), which was calculated as the time from pathological diagnosis to the point of death or the last follow-up record. Survival curves among different patient subgroups were compared with the Mantel log-rank test. The threshold for statistical significance was set at P < 0.05.

## Results

### Patient characteristics

Table 1 summarizes the data of the patients treated at our center, meanwhile the clinical characteristics of all the 35 patients were listed in Table 2. The study group comprised 10 males and 25 females within an age range of 1–56 years old. The most common presenting symptom was perianal/perineal mass, only one patient initially complained of an inguinal mass. Other symptoms including pain, hematochezia, difficult defecation and fecal incontinence (3%) were noted. Fifteen patients had regional lymphatic metastases and 5 patients had distant metastases at the time of diagnosis. The IRS classification was group I in 1, group II in 11, group III in 8 and group IV in 5 cases. The pre-treatment staging was stage-2 in 6, stage-3 in 15 and stage-4 in 5 cases.

**Table 1:** Clinical features in 15 patients from the Sixth Affiliated Hospital and the Cancer Center of Sun Yat-sen University

Gender/age	Presenting symptoms	Misdiagnosis/mistreatment	Time interval from symptom onset to pathological diagnosis	Tumor site	Maximum diameter of tumor (cm)	Treatment*	Pathological subtype	IRS clincial group/stage	outcome	Course of disease/survival time (m/m)
1/ < 10 y	Perianal mass	None	3 d	Perineal	6.6	LRR(R0) + Chemotherapy	ERMS	IV/T2bN1M1(lung, pleura, Mediastinum)	AWD	–/86
2/ ≥ 20 y	Perianal mass	None	1 m	Perineal	4.5	APR(R0) + Chemotherapy	ARMS	II/T2aN1M0	DOD	24/24
2/ ≥ 20 y	Perianal mass	None	1 m	Perineal	12.2	Chemotherapy	ERMS	III/T2bN1M0	DOD	18/18
2/10–19 y	Perianal mass	None	1 m	Perianal	6.8	Chemotherapy	ERMS	IV/T2bN1M1(bone; pelvic; retroperitoneum)	DOD	19/19
2/ ≥ 20 y	Painfulperianal mass	Perianal abscess/abscessotomy	2 m	Perianal	10.0	APR(Rx) + Chemotherapy	ARMS	II/T2bN1M0	DOD	8/8
2/ ≥ 20 y	Painful perianal mass	None	7 d	Perianal	4.7	APR(R0) + Chemotherapy	ARMS	I/T2aN0M0	AWD	12/12
2/10–19 y	Perianal mass	None	2 m	Perineal	5	Inguinal lymph node dissection + chemotherapy	ERMS	III/T2bN1M0	DOD	25/25
2/ ≥ 20 y	Perianal mass	Perianal abscess/ Intravenous antibiotics	2 m	Perianal	8	LRR(Rx) + Inguinal lymph node dissection + CRT	ARMS	II/T2bN1M0	NED	4/96
2/10–19 y	Perianal mass	None	5 m	Perianal	6.0	LRR(R0) + CRT	ERMS	II/T2bN0M0	NED	15/120
1/10–19 y	Perianal mass;; difficult defecation	None	1 m	Perianal	1.8	Chemotherapy	ERMS	IV/T2aN1M1(bone)	DOD	11/11
2/ ≥ 20 y	Perianal mass	Perianal abscess/Abscessotomy + partial mass ectomy	4 m	Perianal	6.2	APR(R0) + Chemotherapy	ARMS	II/T2bN0M0	DOD	11/11
2/10–19 y	Painful perianal mass	Abscessotomy	2 m	Perianal	6.3	LRR(Rx) + CRT	ERMS	II/T2bN1M0	NED	10/24
2/10–19 y	Painful perianal mass; Bloody stool	None	2 m	Perianal	7.5	Chemotherapy	ARMS	IV/T2bN1M1(breast; abdominopelvic)	DOD	11/11
2/10–19 y	Inguinal mass	None	3 m	Perineal	5.9	LRR(Rx) + abdominopelvic and inguinal lymph node dissection + CRT	ERMS	II/T2bN1M0	NED	12/12
2/ ≥ 20 y	Painful perianal mass	Abscessotomy	3 m	Perianal	10.0	Chemotherapy	ARMS	III/T2bN1M0	DOD	11/11

*y* year, *m* month, *d* day, *ARMS* alveolar rhabdomyosarcoma, *ERMS* embryonal rhabdomyosarcoma, *NED* no evidence of disease, *DOD* died of disease, *AWD* alive with disease

*LRR local radical resection, *APR* abdominoperineal resection, *CRT* chemoradiation therapy, *R0* microscopic negative margins, *R1* microscopic positive margins, *Rx* not evaluated

**Table 2:** Data of 35 patients

Age	23 m–56 y (median, 23 y)	
	< 10 y	4 (11%)
	10–19 y	13 (38%)
	≥ 20 y	18 (51%)
Gender	Male	10 (29%)
	Female	25 (71%)
Symptoms	Perianal mass	34 (97%)
	Pain	20 (57%)
	Inguinal mass	2 (6%)
	Bloody stool	2 (6%)
	Difficult defecation	2 (6%)
	Fecal incontinence	1 (3%)
Misdiagnosis history/time interval of diagnosis		35 (3 d–10 m, 3.1 m)
	Misdiagnosis	16 (10 d–3 m, 2.6 m)
	No misdiagnosis	19 (3 d–10 m, 3.4 m)
Pathological subtype	Alveolar	9
	Embryonal	14
	Pleomorphic	1
	ND	11
IRS clinical group	I	1
	II	11
	III	8
	IV	5
	ND	10
Stage	2	6
	3	15
	4	5
	ND	9
Tumor site	Perianal	26
	Perineal	9
Tumor size	< 5 cm	12
	≥ 5 cm	22
	ND	1
Regional lymph node metastasis	N0	7
	N1	15
	ND	13
Distant metastasis	M0	17
	M1	5
	ND	12
Surgical approach	LRR	12
	APR	7
	No surgery or biopsy only	9
	ND	7
Outcome	Survival time < 5 y	5
	NED	3
	AWD	2
	Survival time ≥ 5 y	3
	NED	2
	AWD	1
	DOD	14
	ND	13

*y* year, *m* month, *d* day, *NED* no evidence of disease, *DOD* died of disease, *AWD* alive with disease, *ND* not described

### Imaging

**Fig.1:**
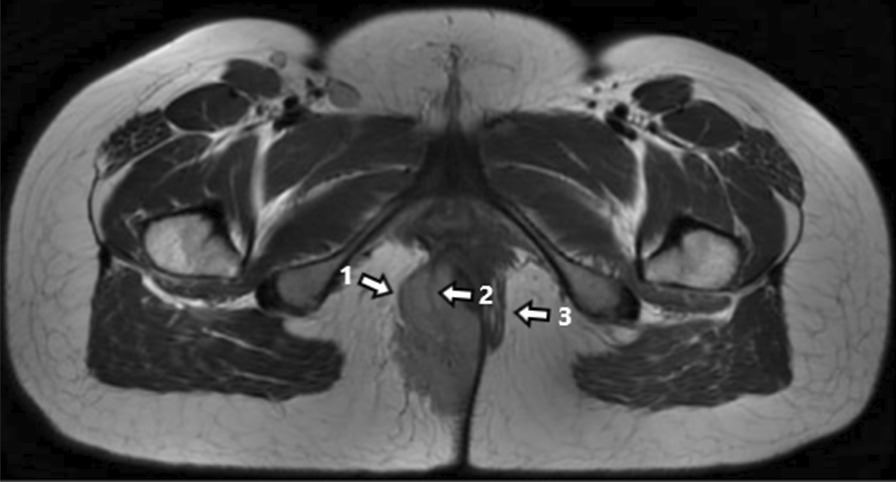
EAS was suppressed and formed Pseudocapsule (arrow 1); Part of the remaining muscle bundles were cutted into the tumor (arrow 2); contralateral EAS remained intact (arrow 3)

**Fig. 2:**
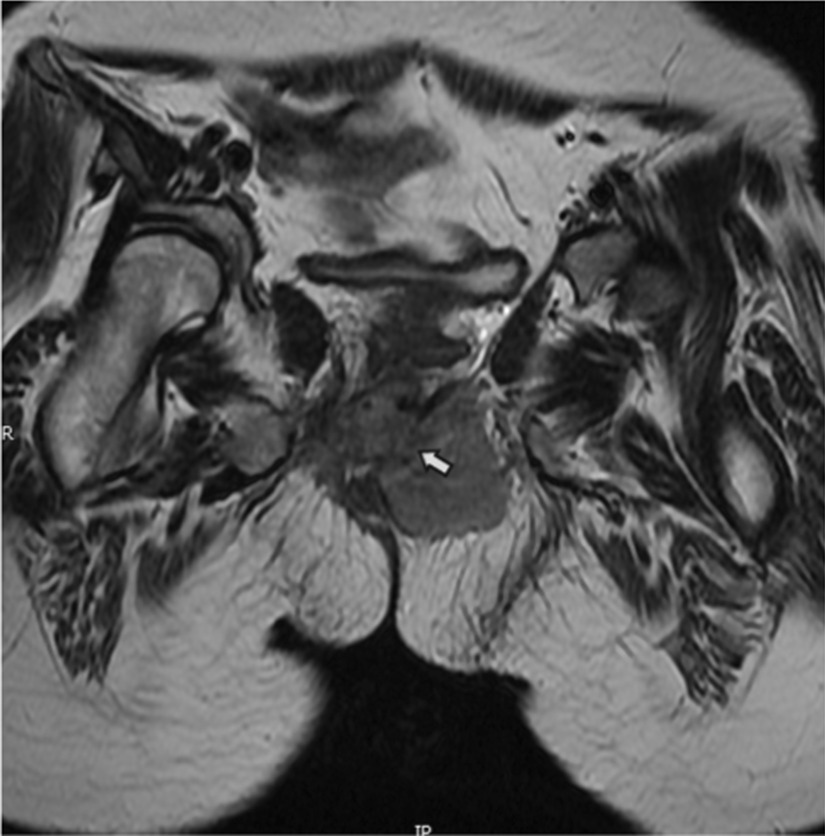
Puborectalis was replaced by tumor signal and bilateral levator ani was involved (arrow)

Fifteen patients treated at our center received imaging evaluation. Four patients were examined by color Doppler ultrasound, which reviewed hypoechoic or mix-echoic masses with unclear boundaries and irregular shapes. Spot blood flow signals were detected by CDFI in 3 cases and 1 case had abundant blood flow signals around the mass. These patients were then subjected to ultrasound-guided biopsy and pathology confirmed the diagnosis of RMS. CT/MRI scans were performed for all the 15 patients, which delineated an average maximum tumour diameter of 6.8 cm (1.8–12.2 cm). Tumour compression of the internal anal sphincter and rectal mucosa was observed in 5 cases and 3 of them had urogenital involvement. MRI was conducted in 10 patients and demonstrated equal signal on T1WI (n = 10) and high (n = 5) or mixed (n = 5) signals on T2WI within tumours. Furthermore, external anal sphincter (EAS) involvement was observed in all of the 15 cases (Fig. 1), including 3 having puborectalis involvement and 3 with levator ani involvement (Fig. 2). Regional lymph node metastases were found in 12 patients. Moreover, 5 patients had distant metastases at distant sites including bones, lungs, pancreas, breasts and the pelvic cavity. Clinical stages were determined based on imaging evaluation as follows: 5 cases in stage-2, 6 in stage-3 and 4 in stage-4.

### Pathological findings

**Fig. 3:**
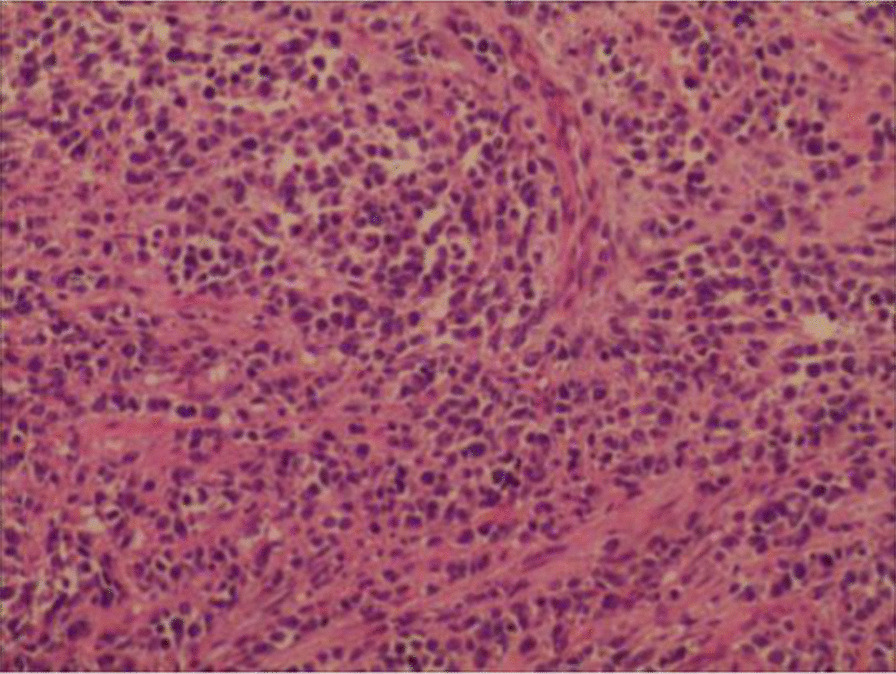
Alveolar RMS: Round, oval or spindle shaped tumor cells were arranged in an acinar, tubular, fissured or nest-like form. Mutifocal necrosis and interstitial fibrosis were constantly presented. (Magnification: × 40)

**Fig. 4:**
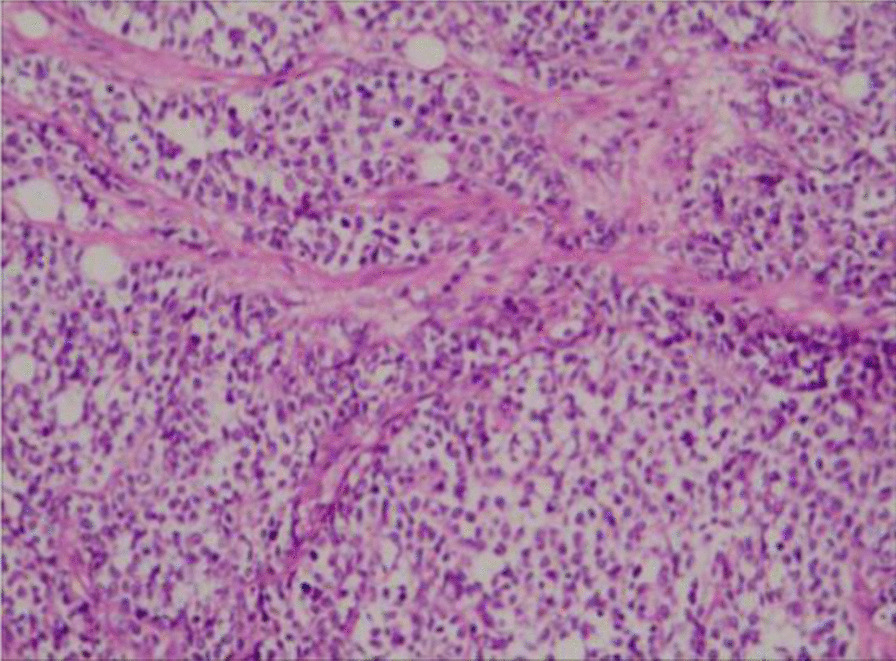
Embryonal RMS was characterized by small round cells with scant eosinophilic cytoplasm and hyperchromatic ovoid-shaped nuclei. (Magnification: × 40)

Six of 15 and 9 cases in our group were histologically confirmed as alveolar RMS (Fig. 3) and embryonal (Fig. 4), respectively. Myogenin was expressed in all cases. Desmin and vimentin were positive in 5 of the 5 cases. MyoD1 was positive in 3 out of 4 cases. Of all the 35 patients, 14 were classified as embryonal RMS, 9 were labeled as alveolar RMS and 1 was identified as pleomorphic RMS. Furthermore, 11 cases had no histological reports. A total of 20 cases had immunohistochemical records reporting the following: Desmin (+) (20/20), vimentin (+) (14/14), myogenin (+) (15/15), MyoD1 (+) (9/10) and myoglobin (+) (9/9).

### Treatments and outcomes

Ten patients received surgical treatment at our center. Primary surgery was performed for 3 patients (R0 = 2, Rx = 1) and secondary surgery after chemotherapy was performed for 6 patients (R0 = 3, R1: n = 1; Rx: n = 2). Among these patients, 4 received an abdominoperineal resection (APR) and 5 received a local radical tumor resection (LRR). One patient only underwent an inguinal lymphadenectomy only. All 15 patients in our center received a chemotherapeutic regimens such as MAID (Mesna, adriamycin, isocyclic amide, dacarbazine), CYVADIC (Cyclophosphamide, vincristine, adriamycin, dacarbazine), CEVAIE (Carboplatin, epirubicin, vincristine, dactinomycin, ifosfamide, etoposide), FOLFOX (Fluorouracil, oxaliplatin, calcium folinate) and TP (Cisplatin, paclitaxel) regimens. Three patients received radiotherapy (RT) postoperatively and one preoperatively. The median survival time was 14 months (ranging from 8 to 120 months). Nine patients died of disease progression after diagnosis (8–25 months).

**Fig. 5:**
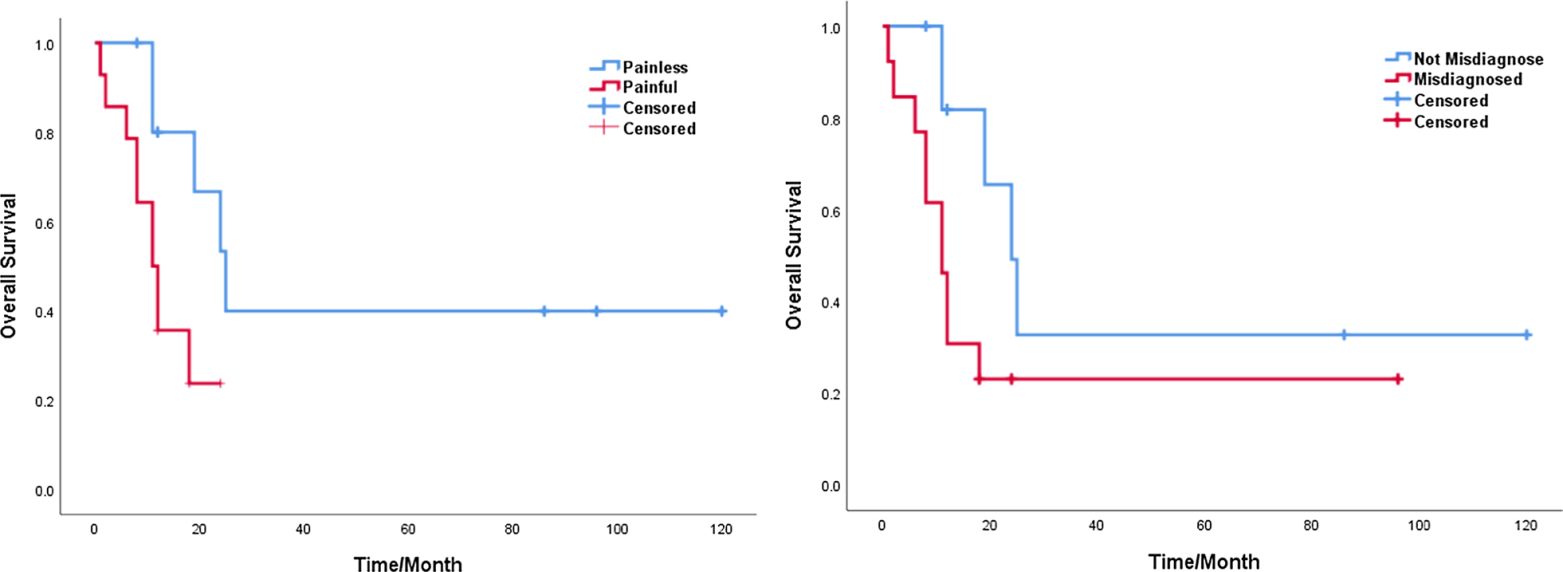
**a** Kaplan–Meier curves comparing survival of patients with or without pain (P = 0.024). **b** Kaplan–Meier curves comparing survival of patients with or without misdiagnosis (P = 0.038)

Taking into account the information retrieved from the Chinese database, 18 out of the 35 patients underwent surgical treatment (APR: n = 7; LRR: n = 11) and 9 of them received radiotherapy or chemoradiotion therapy (CRT) alone. The treatment modality was unknown in 8 cases. Fifteen patients were misdiagnosed as perianal abscesses, 14 of them underwent an abscessotomy and 1 was treated with intravenous antibiotics only. One patient was misdiagnosed as anal fistula with subsequent fistula resection. The presence of pain was found to be related with misdiagnosis (p = 0.010) (Table 3). The average time interval from symptom onset to pathological diagnosis was 3.1 months (3 days to 10 months), 2.6 months for patients with a history of misdiagnosis (10 days to 3 months) and 3.4 months (3 days to 10 months) for those without a history of misdiagnosis. The median survival time was 12 months (Table 2). The 2-year and 5-year overall survival rates were 33% and 25% respectively. The Kaplan–Meier survival analysis showed that the presence of pain (P = 0.024) and misdiagnosis (p = 0.038) were associated with a poor prognosis (Fig. 5).

**Table 3:** Factors of misdiagnosis

Features	Misdiagnosis		P
	No	Yes	
Pain			
No	12	3	0.010
Yes	7	13	
Gender			
Male	5	5	0.519
Female	14	11	
Age			
< 20 y	12	5	0.061
≥ 20 y	7	11	
Tumor site			
Perianal	13	13	0.319
Perineal	6	3	
Tumor size			
< 5 cm	8	4	0.285
≥ 5 cm	11	11	
Pathological subtype			
Alveolar	5	4	0.239
Embryonal	11	3	
IRS clinical group			
I	1	0	0.783
II	7	4	
III	4	4	
IV	4	1	
Stage			
2	5	1	0.391
3	8	7	
4	4	1

## Discussion

RMS is a malignant mesenchymal neoplasm that exhibits striated muscle differentiation, accounting for 5–10% of all solid tumors and 55–60% of soft tissue sarcomas in the pediatric age group, but are relatively rare in adults [20, 21]. However, patients aged ≥ 20 years old accounted for 51% of this retrospective study’s sample size. The most common sites of occurrence for primary RMS were the head/neck, extremities and genitourinary tract [22], with the perianal and perineum area being rare sites of occurrence and considered unfavourable [1, 3, 23]. RMS in adults is more likely to occur in unfavourable sites compared to adolescents [24], which may explain the higher percentage of adults in this study. In addition, female predominance is noticed in this study (71%). Similar features were reported in a Japanese study [25], indicating regional and ethnic differences in the occurrence of this disease.

The prognosis of primary perianal and perineal RMS is extremely poor. Prognostic factors include age, pathological type, clinical group and staging [26]. In this study, patients older than 20 years old comprised 51% of the patient population, 38% were classified as alveolar RMS and 52% were categorized into IRS groups III-IV, which may all contributed to the poorer outcomes. Besides, the rate of misdiagnosis was 45.7% in this group, which was significantly related to a poorer prognosis (p = 0.038).

PRMS cases are frequently misdiagnosed as perianal abscesses, leading to a poor prognosis. Hence, careful evaluation and differential diagnosis for suspected patients are crucial. Perianal masses are the most common manifestation of both PRMS and perianal abscesses. In fact, 97% of patients presented as perianal masses in this study. Perianal abscesses are almost always accompanied by pain [27]. In this study, pain was found to be correlated with the probability of misdiagnosis (p = 0.010). When combined with infection, local redness, swelling and fever can be observed, which resembles the clinical presentation of perianal abscesses and thus leads to a high rate of misdiagnosis [2]. It is difficult to distinguish PRMS from perianal abscesses solely based on symptoms. For firstly-diagnosed patients with painful perianal masses, paying attention to any delay in seeking health care, predisposing factors for abscess formation (Immune deficiency, HIV infection, diabetic ketoacidosis and Crohn's disease) and the increased levels of lactate dehydrogenase would be helpful to establish differential diagnoses [2]. Additionally, epidemiological characteristics can provide clues suggesting that perianal abscesses mainly affect adults and male infants younger than 1 year old [28–30], which is different from the demographic features of PRMS reported in relevant literatures [25, 31, 32]. Empirically, whenever a young woman presents with a perianal mass, especially those under 20 years old, a differential diagnosis of solid or even malignant tumors, including PRMS should be considered. Theoretically, misdiagnosis would delay correct assessment of this disease [2]. However, correlation between misdiagnosis and the average time interval from symptom onset to pathological diagnosis was found statistically insignificant in this study (p = 0.712). On the contrary, the average pathological diagnosis time of misdiagnosed cases was shorter than that of cases without misdiagnosis (2.6 months vs. 3.4 months). A possible reason for this finding may be the fact that patients’ complaints about pain prompted clinicians to carry out emergency abscess incision/resection, rendering an earlier acquirement of postoperative pathological results than those without pain.

Doppler ultrasound is a noninvasive, accessible and radiation-free method for preliminary examination of patients with perianal painful masses suspected to be PRMS. It has unique advantages in distinguishing solid, cystic or lacunar masses, providing high specificity and sensitivity for the diagnosis of perianal abscess. Endoanal ultrasound can clarify tumor involvement of the anal canal, and thus provide clues for accurate diagnosis and adequate treatment [33]. Five cases of PRMS treated at our center revealed solid masses with uneven echo and rich intratumural blood flow signals with or without clear boundaries, which was consistent with the relevant reports [34–36]. One case indicated a perianal abscess by Doppler ultrasound, but the pathological report following a concomitant ultrasound-guided biopsy corrected the diagnosis, which could be implemented as a reliable method to further lower the risk of misdiagnosis. Endoscopic ultrasound-guided fine needle aspiration is recommended for its accuracy and minimal invasion when conducting biopsies [37].

The sonographic features of RMS are variable and nonspecific and have certain limitation on the assessment of deep-tissue lymph nodes and distant metastases [38]. Hence justifying the necessity of CT or MRI for further evaluation. MRI provides clearer soft tissue imaging compared to CT scans and can better reveal the degree of invasion of the RMS in the adjacent pelvic organs. Thus, MRI has become the imaging modality of choice for the diagnosis and evaluation of pelvic RMS, but only a few reports are available for MRI description of PRMS [39–41]. By observing the sagittal, coronal, and axial T2 weighted high-resolution images of the pelvis, we found that the hyposignal of the external anal sphincter (EAS) is replaced by the tumour’s hypersignal in PRMS, causing a discontinuity of the anal sphincter complex. In some cases, the muscle signal of the EAS can be observed cutting into the tumour; a pseudocapsule-like structure was formed by the compressed EAS surrounding the tumour (Fig. 1). According to these features, we suggest that PRMS probably originates from the EAS. Compression rather than direct invasion of the rectal wall and anal canal was observed in 4 cases, which is different from the characteristics of anal canal cancer and thus a valuable clue for differential diagnosis [42, 43].

Among the 15 patients treated at our center, 5 underwent a previous abscess incision or drainage previously at other hospitals and 4 of them had a poor prognosis. For the 2 patients who survived for more than 5 years, one was initially diagnosed as a perianal abscess but only treated with antibiotics without surgery; both of them underwent a radical operation (R0) later on at our center and no evidence of recurrence was found after a 5-year follow up. We noticed that both the presence of pain and a history of misdiagnosis were closely related to poor outcomes. Patient’s complaining of pain usually mislead the clinician’s judgement and impelled him/her to perform the wrong surgical intervention, which may damage the integrity of the tumour and increase the difficulty of radical surgery, thus resulting in an unfavourable prognosis. Nonetheless, as pain itself may indicate local inflammation and progressive infiltration of the malignancy, such signs and symptoms could be considered as risk factors for poor outcomes independent from misdiagnosis. The relationship between pain, misdiagnosis and overall survival requires further verification. Nevertheless, if PRMS are diagnosed on time and distant metastases are excluded, surgical treatment should be carried out as soon as possible. Taking into account the possibility that this tumour likely originates from the EAS, an ELAPE (extra-levator abdominalperineal excision) could be the surgical approach of choice for most patients to achieve a R0 excision. If the tumor was relatively confined and only a small portion of the EAS was involved, a LRR with preserved anus could be performed. As a matter of fact, 5 patients received a LRR at our center and 3 of them survived for more than 5 years; 1 experienced a recurrence due to failure to achieve a R0 resection and is currently undergoing further treatment; 1 has no evidence of recurrence after 2 years of follow-up but the disease course was prolonged due to a rectovaginal fistula caused by surgical damage of the perineum. LRR can achieve long-term survival without compromising the anal function, but a R0 resection must be guaranteed and the perineum should be protected during the operation to avoid complications such as rectovaginal/rectourethral fistulae.

Optimal treatment for PRMS is controversial. Recently, a report from the CWS trials emphasized the role of surgery in the multidisciplinary management of PRMS, with its average 5y-OS reaching 47% [32]. While another study, with a reported average 39% 5y-OS, suggested that patients undergoing surgery in addition to CRT had similar outcomes as those who received only CRT [26]. In this group, the average 5y-OS of patients who underwent radical resection with adjuvant therapy was only 33%, which is lower than those reported by the aforementioned studies. This could probably be due to the lower implementation rate of irradiation. While the North American studies recommended RT in all RMS patients except for those in Clinical Group I ERMS, utilization of RT was more cautious in European trials due to concerns regarding its long-term damaging effects [44]. Without a specific international guideline for RT indication in PRMS, such a measure was only applied in patients who underwent LRR to ensure better local control in our study. In fact, 4 patients who followed this treatment protocol all achieved satisfactory outcomes during our follow-ups, indicating the potential of RT in terms of local control and sphincter preservation [26]. Inguinal lymph node metastasis is one of the main factors of poor outcomes in these patients. In this group, 11 cases were complicated with lymph node metastasis. The average 5-year OS was only 26% for the 9 patients with preoperative inguinal lymph nodes involvement. One patient had ilial lymph node metastasis within 5 months and died in 11 months following surgery. Regarding the high prevalence of regional lymph node involvement in PRMS, it is suggested that inguinal lymph nodes resection or irradiation should be performed prophylactically to control postoperative regional recurrence [26, 31], but such an aggressive approach could be avoided with the application of PET-CT scans which can effectively improve the detection rate of lymph node metastases [45, 46]. In this study, breast metastases were found in 2 cases in this study, which was also reported in a Japanese literature [25], providing insights on the specific metastatic pathway and histological characteristics of PRMS.

This study is mostly limited by its retrospective design, small sample size and the incomplete details from the cases included from Chinese literatures. Given these limitations, an international, large scale, multi-center study should be conducted to validate these data.

## Conclusion

In summary, primary PRMS are rare and easily misdiagnosed soft tissue lesions, which often leads to poor outcomes. Patients presenting with painful perianal masses should be carefully evaluated to exclude this malignancy. PRMS are found to be closely related to the external anal sphincter, and MRI should be conducted to determine EAS invasion. Comprehensive therapy including radical operation, CRT and chemotherapy is often necessary and recommended. It is imperative to achieve further improvements in the clinical outcome of PRMS patients by developing new therapeutic modalities.

## Data Availability

The datasets generated during and/or analyzed during the current study are available from the corresponding author on reasonable request.

## Abbreviations

RMS: Rhabdomyosarcoma
PRMS: Perianal/perineal rhabdomyosarcoma
CT: Computed tomography
MRI: Magnetic resonance imaging
IRS: Intergroup Rhabdomyosarcoma Study
CDFI: Color Doppler flow imaging
EAS: External anal sphincter
APR: Abdominoperineal resection
LRR: Local radical tumor resection
MAID: Mesna + adriamycin + isocyclic amide + dacarbazine
CYVADIC: Cyclophosphamide + vincristine + adriamycin + dacarbazine
CEVAIE: Carboplatin + epirubicin + vincristine + dactinomycin + ifosfamide + etoposide
FOLFOX: Fluorouracil + oxaliplatin + calcium folinate
TP: Cisplatin + paclitaxel
